# Time-restricted feeding delays the emergence of the age-associated, neoplastic-prone tissue landscape

**DOI:** 10.18632/aging.102021

**Published:** 2019-06-12

**Authors:** Monica Serra, Fabio Marongiu, Maria Giuseppina Pisu, Mariangela Serra, Ezio Laconi

**Affiliations:** 1 Department of Biomedical Sciences, University of Cagliari, Cagliari, Italy; 2 Neuroscience Institute, National Research Council of Italy (CNR), Cagliari, Italy; 3 Department of Life and Environment Sciences University of Cagliari, Cagliari, Italy

**Keywords:** time restricted feeding, carcinogenesis, aging, tissue microenvironment

## Abstract

Aging increases the risk of cancer partly through alterations in the tissue microenvironment. Time-restricted feeding (TRF) is being proposed as an effective strategy to delay biological aging. In the present studies, we assessed the effect of long-term exposure to TRF on the emergence of the age-associated, neoplastic-prone tissue landscape. Animals were exposed to either *ad libitum* feeding (ALF) or TRF for 18 months and then transplanted with hepatocytes isolated from pre-neoplastic nodules. Both groups were continued ALF and the growth of transplanted cells was evaluated 3 months later. A significant decrease in frequency of larger size clusters of pre-neoplastic hepatocytes was seen in TRF-exposed group compared to controls. Furthermore, TRF modified several parameters related to both liver and systemic aging towards the persistence of a younger phenotype, including a decrease in liver cell senescence, diminished fat accumulation and up-regulation of SIRT1 in the liver, down-regulation of plasma IGF-1, decreased levels of plasma lipoproteins and up-regulation of hippocampal brain-derived growth factor (BDNF).These results indicate that TRF was able to delay the onset of the neoplastic-prone tissue landscape typical of aging. To our knowledge, this is the first investigation to describe a direct beneficial effect of TRF on early phases of carcinogenesis.

## Introduction

The link between aging and neoplastic disease is widely acknowledged. In addition, it is increasingly apparent that some of the most relevant risk factors for cancer in humans are also associated with signs of accelerated aging in their target organ. Examples include smoking for the respiratory mucosa, chronic hepatitis for the liver, UV light for the skin, chronic (atrophic) gastritis for the stomach, among others [1–7]. This underscores the argument that the pathophysiology of aging is strictly intertwined with that of carcinogenesis, to the point that the emergence of the aged phenotype stands as a major biological driving force towards neoplastic development. Nevertheless, this association is still awaiting a fully satisfactory mechanistic explanation.

In approaching this issue, transplantation studies conducted by our research group revealed that the microenvironment of the aged rat liver is permissive for the clonal growth of both normal and pre-neoplastic hepatocytes, while the same cells, either normal or pre-neoplastic, do not expand when seeded in the liver of young recipients [8,9]. Furthermore, pre-neoplastic hepatocytes were able to progress to form large tumors in aged hosts, while no lesions were detected in animals transplanted at young age even after several months of follow-up [9]. Based on this evidence, we have proposed that a relevant contribution of aging towards increasing the risk of cancer is related to the emergence of an age-associated, clonogenic and neoplastic-prone tissue landscape [10], the declining cellular fitness of the aged tissue being a selective driver for clonal growth, as it has been suggested for the hematopoietic system [11,12].

While the aging process *per se* is largely unavoidable, the rate of aging is amenable to modulation. Decades of studies have indicated that dietary control is among the most effective strategies to delay aging and age-related diseases, including cancer [13]. Most consistent results have been obtained with caloric restriction, i.e. a reduction of total daily calories without causing malnutrition. Both lifespan and healthspan extensions were reported in virtually all species that were fed a caloric-restricted diet, although some controversies do exist regarding the interpretation of data obtained in non-human primates [14]. However, practical difficulties and theoretical concerns limit the applicability of caloric restriction regimens to humans [15]. An alternative approach that is being actively explored for its potential benefits is time-restricted feeding (TRF) [16]. In this dietary protocol food consumption is not randomly distributed across the 24 hours, but it is limited to a daily interval of 8 to 12 hrs, while the total amount of ingested calories is comparable to that of *ad libitum* fed (ALF) controls. The main rationale of TRF regimen is that the time of food intake should be aligned with internal circadian rhythms, in order to synchronize with tissues metabolic needs [17]. An increasing number of studies have indeed indicated that TRF is able to reproduce at least some of the effects associated with caloric restriction, particularly on metabolism [18]. Thus, TRF was shown to prevent excess increase in body weight both in *Drosophila* *melanogaster* [19] and in mice fed either regular or high fat diets [20]. Moreover, TRF could also reverse pre-existing diet-induced obesity, both after long and short-term exposure [16,21], as well as diet-induced liver steatosis and the accompanying increase in serum markers of liver disease [22], while plasma levels of triglycerides and low density lipoproteins were decreased [16,22]. A beneficial effect of TRF on the emergence of insulin resistance has also been reported in young and middle-aged mice [16,20]. Most recently, a TRF regimen was also shown to increase lifespan in mice [23].

Limited evidence is available so far on the possible impact of TRF on neoplastic disease. A retrospective epidemiological study in humans reported a positive correlation between protection from breast cancer risk and duration of overnight fasting period [24]. In a more recent report, exposure to TRF for 8 weeks was found to mitigate high-fat diet-enhanced mammary tumorigenesis in the mouse mammary tumor virus-polyoma middle tumor-antigen (MMTV-PyMT) model [25]. Based on these premises, the present investigation analyzes the effect of long term TRF on the emergence of the age-associated, neoplastic-prone tissue microenvironment in rat liver. Furthermore, the possible effect of TRF on liver specific and systemic, age-related phenotypic alterations is also addressed.

## RESULTS

### Food consumption and growth curves

**Figure 1:**
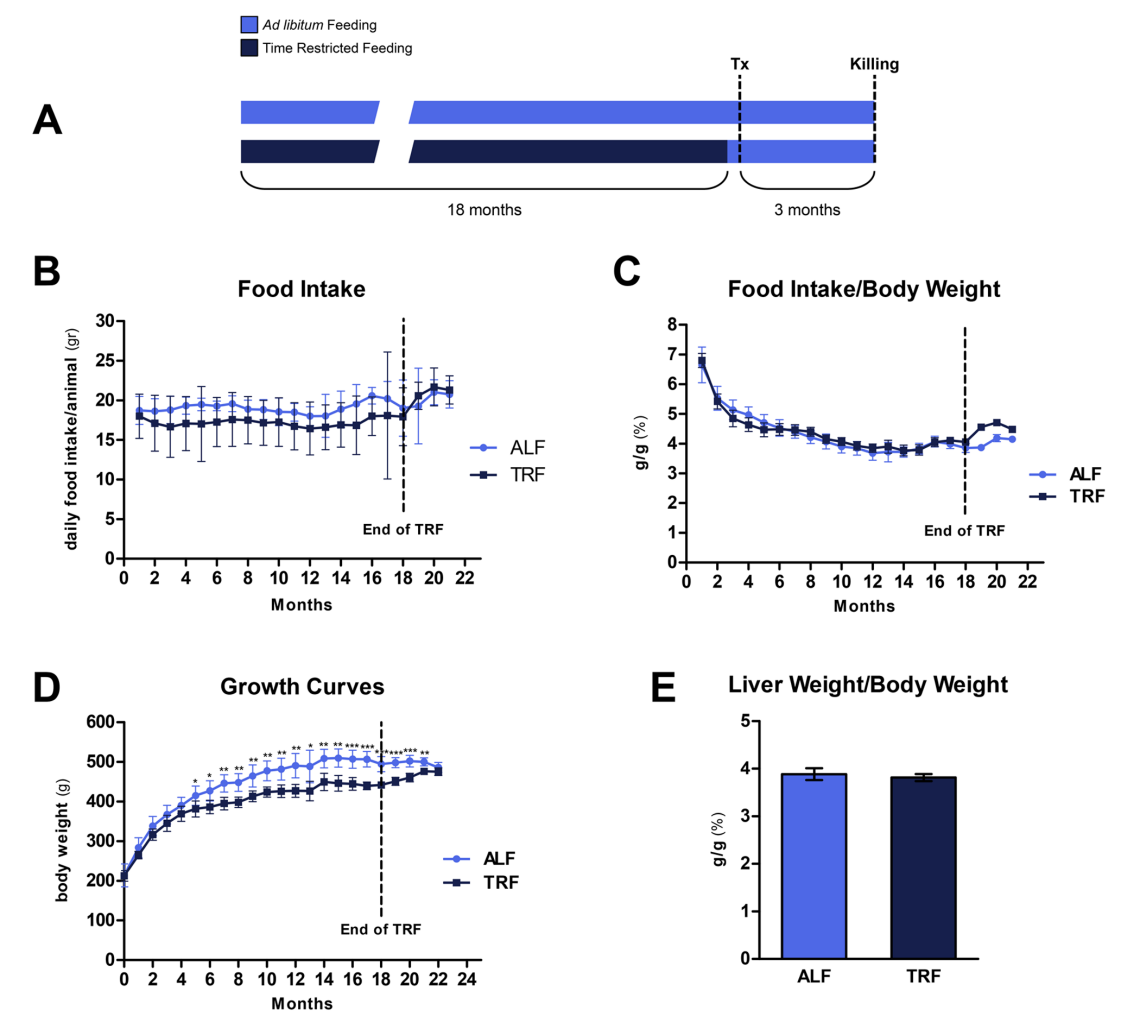
**Schematic representation of experimental protocol** **(A)**. Food intake (**B** and **C**), growth curves (**D**) and relative liver weights (**E**) in animals from ALF or TRF groups. Significant differences between ALF and CRF groups are indicated where appropriate (*p <0.05, **p <0.01, ***p <0.001).

Animals were assigned to TRF or ALF group at 8 weeks of age and they were maintained on their respective dietary regimens for 18 months (Figure 1A). When computed over the entire period, average daily food intake was 19.1±0.7 and 17.2±0.5 for ALF and TRF groups, respectively. However, during the last 3 months of the study, when all groups were fed *ad libitum*, food consumption in TRF group slightly exceeded that of controls (21.2±0.6 vs. 20.4±0.9 g/rat/day, respectively) (Figure 1B).

Interestingly, when food intake was normalized per body weight, no significant differences were observed throughout the 18 months of exposure to either dietary regimens (Figure 1C).

Body weight was monitored weekly for the entire duration of the experiment (Figure 1D). Animals given TRF regimen showed a slightly lower weight gain compared to controls fed *ad libitum*; the difference became statistically significant after about 5 months and persisted thereafter, until 18 months, when it was 89% of controls. However, during the last 3 months, when TRF group was fed *ad libitum*, body weight increased and reached values similar to controls. Relative liver weights at the end of the experiment were not significantly different in the two groups (Figure 1E).

### The growth of transplanted pre-neoplastic cells

In order to evaluate whether TRF had any effect on the onset of the neoplastic-prone tissue microenvironment typical of the aged liver [10], animals were exposed to this dietary regimen for 18 months. They were then switched to ALF and they were transplanted with hepatocytes isolated from chemically-induced hepatic nodules (See Methods for details). Three months later they were killed and the growth of pre-neoplastic hepatocytes was assessed. Livers were excised and analyzed both macroscopically and histologically.

A few visible hepatocyte nodules were discerned on gross examination; their incidence is reported in Table 1. While numbers were insufficient for statistical analysis, a trend towards a decrease was seen in TRF group.

**Table 1:** Liver lesions in rats transplanted with pre-neoplastic hepatocytes.

Nutritional behaviour	Total animals with lesion	Total lesion per group	Size range
*Ad Libitum Feeding*	4/4	8	2 mm
*Time-Restricted Feeding*	3/6	3	1.5-4 mm

**Figure 2:**
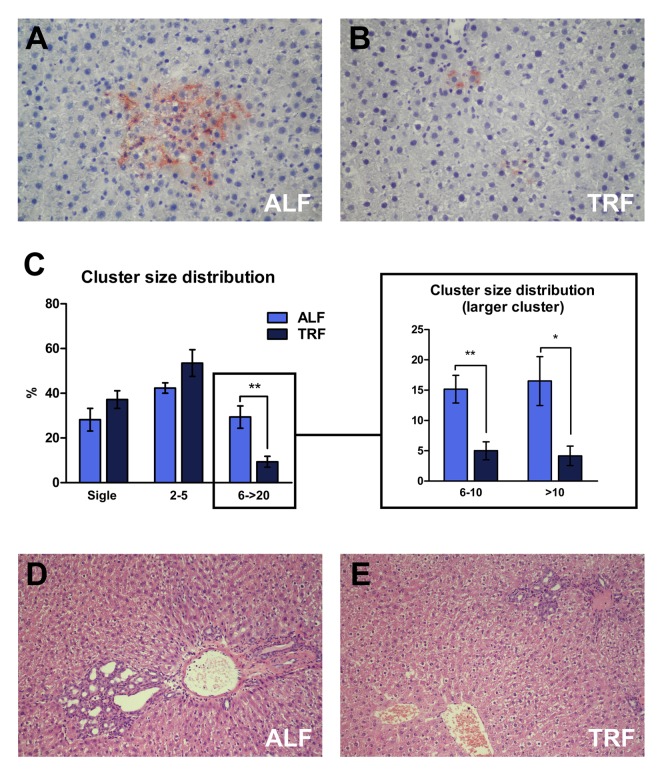
**The growth of transplanted pre-neoplastic hepatocytes in the liver of rats exposed to ALF or TRF regimens for 18 months.** Following transplantations, animals from both groups were continued on ALF for 3 months (see Methods for details). Larger class clusters of DPP-IV expressing, pre-neoplastic hepatocytes (**A**, **B** and **C**) were significantly more frequent in ALF compared to TRF group (*p <0.05 and **p <0.01). (**D** and **E**) H&E staining of livers from ALF or TRF groups, respectively. Note the presence of cholangio-fibrosis, more prominent in ALF group. Magnification: **A** and **B**, 200x; **D** and **E**, 100x.

Histochemical analysis was performed in order to detect DPPIV-expressing, transplanted pre-neoplastic hepatocyte clusters (Figure 2A and B). Ten sections per animal were considered. No significant differences were observed in the total number of clusters/unit area between the two groups, as expected. Percent cluster size distribution in each group is reported in Figure 2C. Singlets, i.e. single DPPIV-positive hepatocytes, added up to 28.2±12.4 and 37.1±9% of all clusters in ALF and TRF groups, respectively. Small clusters, i.e. comprising 2 to 5 cells per cross section, were 21.1±13.0 and 26.8±18.2% of the total in ALF and TRF groups, respectively; finally, larger clusters, i.e. comprising a minimum of 6 and up to 20 cells, were 9.9±7.6 and 3.1±3.6% in ALF and TRF groups, respectively. Thus, larger clusters were about 3-times as frequent in ALF compared to TRF group and these differences were statistically significant (Figure 2C).

Immuno-histochemical detection of Ki67 revealed very low levels of labelling (<0.1%) in both transplanted hepatocyte clusters and surrounding host liver, with no intergroup differences between ALF and TRF treated animals.

Histological analysis of liver samples with standard H&E staining revealed the presence of prominent cholangio-fibrosis and bile ductular proliferation, which have long been described in the aging liver [26]. They were a more frequent finding in rats fed *ad libitum* throughout the experiment (Figure 2D and E).

### Time-restricted feeding and the aging liver

We next investigated whether TRF regimen had any effect on the appearance of phenotypic alterations that have been associated with the aging process in the liver.

### Cell senescence

**Figure 3:**
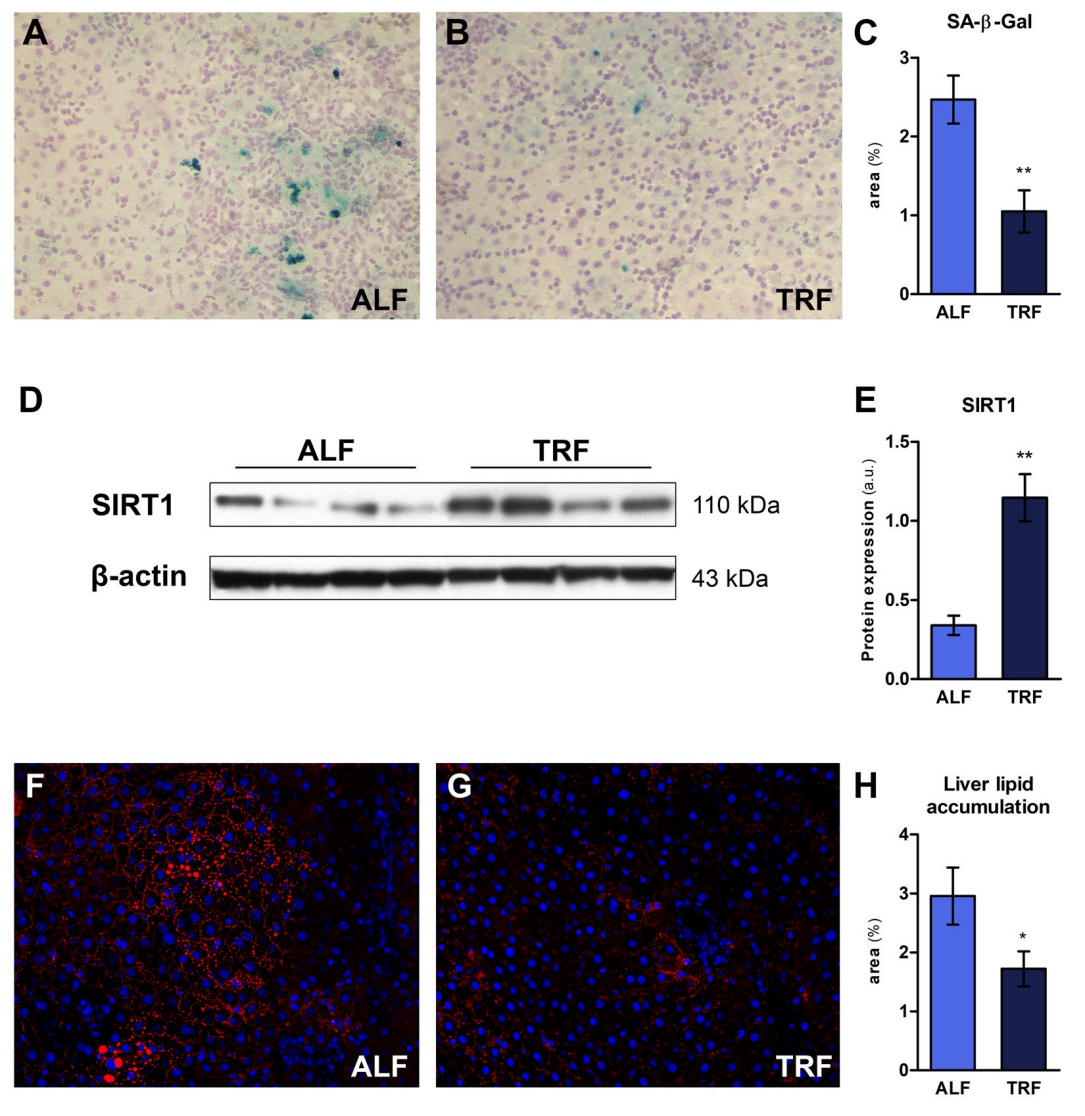
**The effect of TRF on markers of liver aging in rats exposed to ALF or TRF for 18 months and continued on ALF for additional 3 months.** (**A**, **B** and **C**) SA-β-gal staining. Percent area positive for SA-β-gal staining was decreased in TRF compared to ALF group (**p- value <0.01). (**D** and **E**) Western blot analysis of SIRT1 expression. A significant up-regulation was seen in TRF group (**p-value <0.01). (**F**, **G** and **H**) Immunofluorescence analysis of intracellular lipid accumulation (red) in the liver. Nuclei were stained with Hoechst (blue). A decrease in lipid droplets was evident in TRF group (*p- value <0.05).

Cell senescence entails a persistent/irreversible arrest of the cell cycle associated with distinct phenotypic changes [27]. Senescent cells increase with age in many tissues, including liver, although their precise mechanistic role in the overall aging process is yet to be defined, if any [28]. The enzyme senescence-associated β-galactosidase (SA-β-Gal), located in the lysosomal compartment, is the most widely used biomarker for senescent cells [29]. We performed histochemical staining of liver sections obtained from ALF or TRF groups, treated as described in the previous paragraph (18 months on ALF or TRF regimens, followed by 3 months of ad libitum diet). Three sections from each lobe were processed and expression of SA-β-Gal was estimated using an image analyzer (Figure 3A and B). As reported in Figure 3C, a significant decrease in percent SA-β-Gal-positive areas/total was found in TRF group compared to controls.

### Nutrient sensing pathway

Alterations in nutrient sensing pathways are considered as one of the “hallmarks” of aging, and several lines of evidence suggest that such altered metabolic regulation does in fact represent a candidate driver of the aging process [30]. An important role in this context is played by sirtuins, a family of NAD-dependent protein deacetylases involved in diverse mechanisms associated with the emergence of the aging phenotype. Overexpression of SIRT1, a member of the sirtuin family, has been associated with increased longevity and/or healthy ageing, possibly via interaction with major nutrient sensing pathways of longevity, such as mTOR, FOXO and insulin-IGF-1 signaling network [31]. Furthermore, the beneficial effects of caloric restriction on metabolism have been attributed, at least in part, to up-regulation of Sirt1 [32,33]. We therefore investigated whether long-term exposure to TRF was able to modify the expression of SIRT1 in the liver. As shown in Figure 3D and E, a 3-fold increase in SIRT1 protein levels was indeed found in TRF group compared to controls.

### Fat accumulation

Aging is an important risk factor for development of hepatic steatosis [34]. In order to further explore the effect of long term TRF on the emergence of age-associated phenotypes in the liver, the extent of hepatic lipid accumulation was determined in animals exposed to this dietary regimen for 18 months, followed by 3 months of ALF. Cryostat sections obtained from ALF or TRF-treated rats were stained with HCS LipidTOX™ Red phospholipidosis detection reagent (Figure 3F and G). Quantitation of lipid droplets revealed a significantly decreased accumulation in the livers of rats exposed to TRF (Figure 3H).

### Time-restricted feeding and systemic aging

We then explored the effect of long-term exposure to TRF on the emergence of age-associated biochemical and metabolic alterations at systemic level.

### Plasma IGF-1

**Figure 4:**
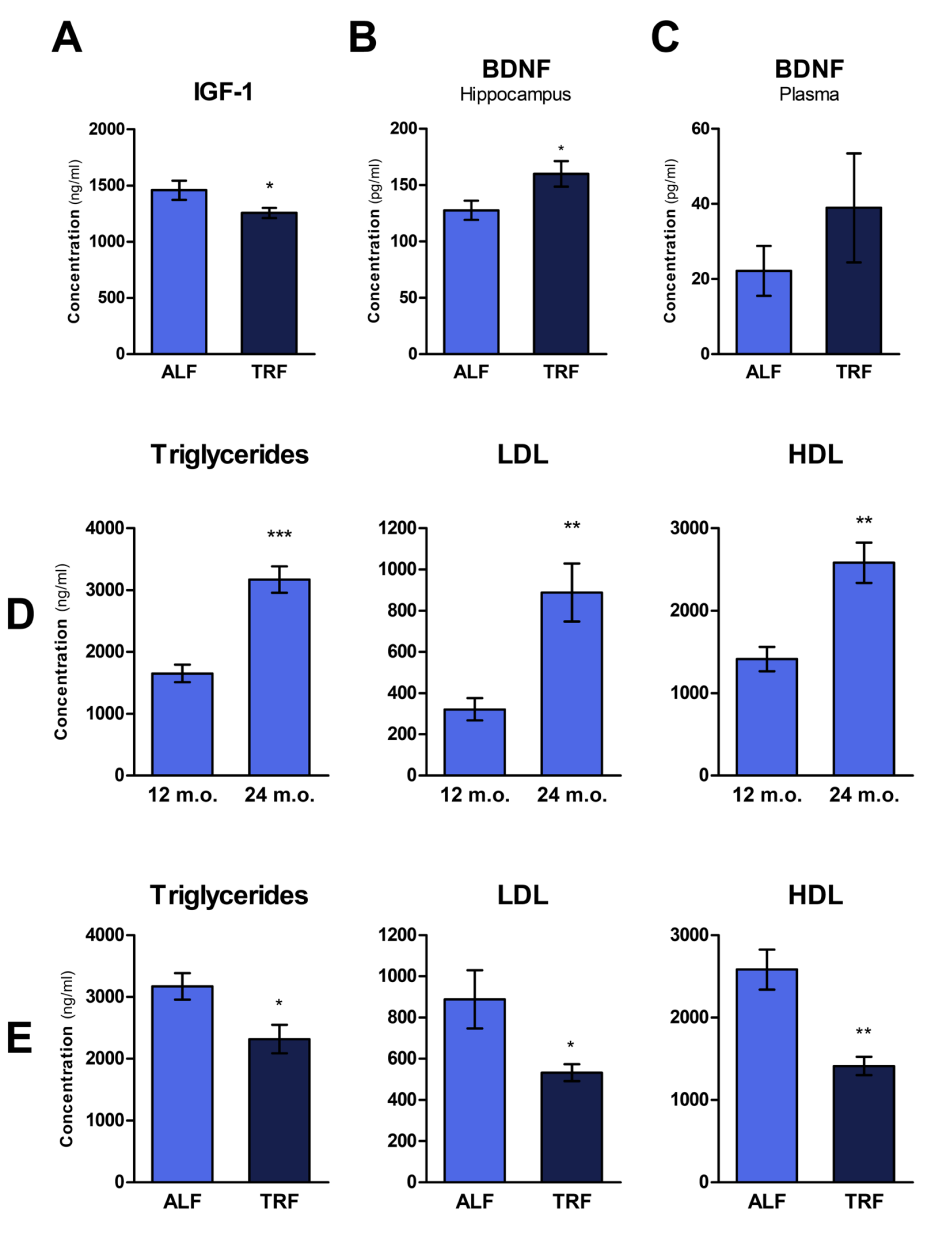
**The effect of TRF on markers of systemic aging in rats exposed to ALF or TRF for 18 months and continued on ALF for additional 3 months.** (**A**) Plasma levels of IGF-1 were decreased in TRF group (p-value <0.05). (**B** and **C**) BDNF levels in the hippocampus and plasma; differences were statistically significant in the hippocampus (*p-value <0.05). (**D**) Plasma triglycerides, LDL and HDL levels in rats after 1 or 2 years on ALF regimen. A significant increase in all parameters was seen at 2 years (**p <0.01; ***p <0.001). (**E**) Plasma lipids profile in rats exposed to ALF or TRF for 18 months and continued on ALF for additional 3 months. Rats receiving TRF had lower levels of triglycerides, LDL and HDL (*p-value <0.05; **p-value <0.01).

Circulating levels of insulin-like growth factor 1 (IGF-1) have been inversely correlated with lifespan in several strains of mice, supporting the hypothesis that its pathway plays a key role in regulating longevity [35]. Accordingly, drugs that prevent IGF-1 release or its binding to the membrane receptor are being proposed as a means to extend lifespan [36]. Using ELISA method, we determined plasma levels of IGF-1 in rats treated with ALF or TRF dietary regimen for 18 months, and then continued on ALF for 3 months. As reported in Figure 4A, significantly lower levels of IGF-1 were detected in TRF group compared to controls.

### Brain derived neurotrophic factor (BDNF) 

Aging also entails a progressive decline of cognitive functions. Brain-derived neurotrophic factor (BDNF) plays an important role in memory formation, neuron plasticity and cell proliferation. Considering the decreased levels of BDNF in the ageing hippocampus, several studies have suggested a mechanistic role for this neurotrophic factor in age-related hippocampal dysfunction, memory weakening and increased risk of neurodegenerative diseases [37]. Most recently, BDNF has also been linked to a host of beneficial effects at systemic level, including decreased adiposity, improved glucose tolerance and reduced steatosis [38]. Thus, to gain further insights into the effect TRF on the aging phenotype, levels of BDNF were measured in plasma and in the hippocampus of rats under the specific experimental conditions of our studies. Plasma and hippocampal cell lysate were analyzed for BDNF peptide. Higher levels of BDNF were observed in both the hippocampus and in plasma of animals exposed to TRF, albeit differences were statistically significant only in the hippocampus (Figure 4B and C).

### Blood lipid profile

Changes in plasma lipid profile are a frequent finding during aging (as also observed in these studies, Figure 4D) and are implicated in the pathogenesis of most common human chronic diseases, such as cardiovascular disease [39]. Recent data suggest that TRF might represent a reasonable and viable strategy to counter the age-associated rise in the incidence of dyslipidemia [22,40]. We tested this possibility under our experimental conditions: plasma lipids, including triglycerides (TG), low density lipoproteins (LDL) and high density lipoprotein (HDL), were measured in rats exposed to ALF or TRF for 18 months, followed by ALF for an additional 3 months. As reported in Figure 4E, TRF treatment was associated with significantly decreased levels of all lipid parameters, in agreement with results of previous studies [16]. Since concentrations of plasma lipids increased with age in control ALF group (Figure 4D), the lowering effect of TRF on these parameters is more correctly interpreted as a delay in their age-associated increase.

## DISCUSSION

Taken together, the results of the present studies indicate that long-term exposure to TRF retards the onset of the clonogenic and neoplastic-prone hepatic tissue microenvironment associated with aging. Furthermore, several age-related alterations were countered by TRF both in the liver and at systemic level.

Using an experimental protocol based on syngeneic cell transplantation, hepatocytes isolated from chemically-induced liver nodules were injected in recipient rats following 18- month exposure to TRF regimen. Animals were then continued on ALF diet in order to avoid a direct effect of TRF on the rate of growth of transplanted cells. Clusters size was evaluated 3 months later. A significant decrease in the frequency of larger size clusters of pre-neoplastic hepatocytes was seen in TRF-exposed group compared to controls given ALF throughout the experiment. This indicates that TRF regimen was able to delay the emergence of the clonogenic and neoplastic-prone tissue soil typical of aging. It is worth noting that the present results parallel very closely those reported by our research group following exposure to caloric restriction. It was shown that the retarding effect a calorie-restricted diet on neoplastic development was attributable, to a large extent, to a decreased clonogenic potential of the liver microenvironment exposed to caloric restriction [41].

The clonogenic drive of aged, phenotypically normal tissues has been highlighted in recent years by several studies indicating that clonal expansions are a frequent finding in old healthy individuals (including humans), to the point that they should come to be considered as a normal, and in fact a universal marker of advancing age [42,43]. Since such clonal expansions include (but are not limited to) cell populations with pre-neoplastic potential (e.g. carrying mutations associated with cancer [42,43]), it follows that the proneness to foster clonal expansions of the aged tissue microenvironment constitutes a risk factor for the emergence of neoplastic disease [44].

To our knowledge, this is the first investigation to describe a direct beneficial effect of long term TRF on early phases of carcinogenesis. It was recently reported that a time-caloric restricted diet could inhibit progression from cirrhosis to hepatocellular carcinoma induced by chronic administration of diethylnitrosamine [45]. However, feeding time in this study was reduced to 2 hours, resulting in a sizeable (~30%) decrease in daily caloric intake. Interestingly, epidemiological studies in women have suggested that prolonged nightly fasting may decrease the risk of breast cancer [46] and breast cancer recurrence [24]. Most recently, exposure to TRF for 8 weeks was found to mitigate high-fat diet-enhanced mammary tumorigenesis in the MMTV-PyMT mouse model [25].

Evidence was also obtained in our studies that TRF is able to delay the onset of age-associated phenotypic alterations, extending previous findings published in the literature [18,20]. Parameters related to both liver and systemic ageing were in fact found to be modulated by TRF towards the persistence of a younger phenotype, including a decrease in liver cell senescence, lower incidence of cholangiofibrosis, diminished fat accumulation and up-regulation of SIRT1 in the liver, down-regulation of plasma IGF-1, decreased levels of plasma lipoproteins and up-regulation of hippocampal BDNF. Of note, the beneficial effect of TRF on the above parameters was still detectable after 3 months of ALF diet, suggesting that it reflects persistent biological adaptations as opposed to transient metabolic alterations.

The finding that TRF is effective in delaying aging phenotypes has two major implications. Firstly, from a practical point of view TRF stands as a dietary manipulation that is more amenable to implementation in humans compared to caloric restriction [16]. Moreover, caloric restriction can carry important side effects such as infertility, depression, osteoporosis, slower wound healing and others [15]. In fact it is well known that dietary restriction in adults may decrease reproductive potential in both sexes, possibly due to re-allocation of resources from reproduction to survival [47,48], while in young and growing animals it is associated with a reduced skeletal bone acquisition, resulting from suppression of bone formation and activation of bone resorption [49]. Since TRF does not imply a (major) reduction in total calories, the above contraindications do not apply.

Secondly, as a follow up to the latter consideration, if several effects of CRF can be reproduced by TRF with no decrease in caloric intake, the implication is that the beneficial effects of the former are mediated, at least in a relevant part, by biochemical/molecular mechanisms set in motion by the latter [23]. Thus, there is a need for new mechanistic hypotheses to be pursued as to the aging-retarding effects of TRF and CRF. Placing emphasis on the time component of feeding behavior, as opposed to the amount of food per se, brings attention to the interplay of the pattern of food ingestion with peripheral circadian clocks and to their relation to the rate of aging [50,51]. This is in line with the increasing awareness in the literature of the concept of “chrono-nutrition”, i.e. the notion that food consumption should be aligned and resonate with body’s daily rhythms in order to prolong healthspan [50]. The underlying implication is that a better understanding and fine-tuning of this reciprocal interaction has the potential to widen our opportunities for effective interventions towards delaying ageing.

As a note of caution, we are aware of the fact that, in our studies, animals exposed to TRF had also a significant, albeit limited component of CRF, since food consumption was about 10% less, on average, in TRF compared to ALF group. While such a small difference in food intake would appear unlikely to be effective in retarding aging unless it is coupled with a TRF regimen, such a possibility cannot be ruled out at present and deserves further investigations.

In summary, the present studies provide the first direct evidence that a TRF regimen is able to delay the emergence of the neoplastic prone/clonogenic tissue landscape associated with ageing. This specific outcome is part of a more generalized beneficial effect of TRF on several biological, biochemical and metabolic parameters related to the aging phenotype.

## METHODS

### Animals and diet

Experiments were performed using Fischer 344 male rats. Animals were maintained on an alternating 12 hours light/dark cycle (light phase from 7AM to 7PM), in a temperature and humidity-controlled environment, with water available ad libitum and housed two for each cage. They were fed a standard rodent laboratory chow diet (Mucedola, Settimo Milanese, Italy, #4RF21). Animal studies were reviewed and approved by the Institutional Animal Care and Use Committee of the University of Cagliari (Italy) and were performed in accordance with the relevant guidelines and regulations. Eight weeks old male rats were randomly divided into 2 groups of 10 animals each: *ad libitum* feeding (ALF) group, which had free access to food throughout the experiment; time-restricted feeding group (TRF), which had access to food for 8 hours during the dark phase, starting at 11PM until 7AM. Body weight was measured weekly whereas food consumption was measured daily for TRF and weekly for ALF. Such feeding regimens were maintained for 18 months. All animals were then continued on ALF diet for an additional period of 3 months. One week after the latter dietary change, both groups were transplanted with pre-neoplastic hepatocytes isolated from syngeneic donor (see below). Three months after transplantation, animals were killed and blood, liver and brain tissue were collected for analysis.

### Generation of hepatic nodules and isolation of pre-neoplastic hepatocytes

To follow the fate of donor pre-neoplastic hepatocytes into the recipient liver, the dipeptidyl peptidase type IV (DPPIV)-deficient rat model was used [9]. Hepatocyte nodules were induced in DPPIV expressing animals according to a modified version of the Solt and Faber protocol. Nine to ten months after treatment, livers were perfused according to a standard 2-step collagenase perfusion technique. Cell viability, determined by trypan blue dye exclusion, was 90-95%. Pre-neoplastic cells (6x10^5^ cells/animal) were transplanted into the liver of DPPIV-deficient syngeneic recipients, via a mesenteric vein. All procedures were performed as previously reported [9].

### Histological analyses

Standard histological analysis was performed on formalin-fixed paraffin-embedded tissue sections after H&E staining. To follow the fate of transplanted cells, histochemical staining for the detection of DPPIV was performed on 5 µm-thick frozen sections as previously described [9]. Staining for SA-β-gal was performed on 7 µm-thick cryostat sections according to published procedures [52]. Liver tissue lipid accumulation was assessed on 5 µm-thick frozen sections using HCS LipidTOX™ Red phospholipidosis detection reagent (Thermo Fisher Scientific, Waltham, MA, USA, #H34351). Immunohistochemical staining for Ki67 was carried out on 5-µm thick frozen sections fixed in 0.1% acetic acid/ethanol, washed in PBS and blocked with goat serum. Primary antibody (Abcam, Cambridge, MA, #ab16667) was applied overnight at 4 °C; detection of specific signal was accomplished using an alkaline phosphatase (AP)-conjugated secondary antibody system (Vectastain ABC and substrate kits; Vector Labs, Burlingame, CA, #AK-5001 and #SK-5300).

### Western blot analysis

Western blot analysis was performed on nuclear proteins extracted from liver samples using a commercially available kit (NE-PER™, Thermo Fisher Scientific, #78835). Protein concentration was measured using the Bicinchoninic Acid Kit (Sigma-Aldrich, Saint Louis, MO, USA, #BCA1). Samples were prepared in Laemmli buffer, boiled at 95°C for 5’, loaded into SDS-PAGE precast Criterion TGX Stain-Free gels (Bio-Rad, Hercules, CA, USA) and run under denaturing conditions. Proteins were transferred onto PVDF membranes, blocked with 5% non-fat milk for 45’ at room temperature, followed by incubation with primary antibodies overnight at 4°C (SIRT1: Santa Cruz Biotechnology, Dallas, TX, USA, #SC-15404 1:1000; β-actin: Abcam, Cambridge, UK, #ab8227 1:10000). Membranes were then washed and incubated for 2 h at room temperature with secondary antibody conjugated with HRP (anti-rabbit IgG: Abcam, #ab205722 1:10000). Protein bands were detected using a chemiluminescent substrate (Bio-Rad, #1705061) and imaged onto Kodak film.

### Blood and brain tissue analyses

Concentration of plasma lipids was measured using commercially available reagents, according to manufacturer’s instructions (HDL and LDL, Sigma-Aldrich, #MAK045; Triglycerides, Sigma-Aldrich, #MAK266).

Sandwich ELISA was performed on plasma samples for the quantitative analysis of target proteins using commercially available kits, according to manufacturer’s instructions. Adiponectin: R&D Systems, Minneapolis, MN, USA, #RRP300; Leptin: BioVendor, Brno, Czech Republic, #RD291001200R; IGF-1: Medignost, Reutlingen, Germany, #E25; BDNF Merck, Darmstadt, Germany.

Sandwich ELISA was also performed on total protein extracts from the hippocampus, for the analysis of BDNF, using kit #RAB1138 (Sigma-Aldrich).

Final absorbance was read on a microplate reader (Infinite F200 pro, Tecan, Männedorf, Switzerland).

### Image analysis, graphical representation of results and statistical analysis

Acquired microscopic images were processed for quantitative analysis using Image-Pro Premier software (Media Cybernetics, Rockville, MD, USA). Western blots were densitometrically quantified with ImageJ software (NIH, USA). Graphical representation of quantitative results and statistical analysis were performed using Prism 5 software (GraphPad Software, La Jolla, CA, USA).

### Data availability

The data that support the findings of this study are available from the authors upon reasonable request and with permission of the Institutional Review of the University of Cagliari.
